# Interleukin-13 −1112 C/T Promoter Polymorphism Confers Risk for COPD: A Meta-Analysis

**DOI:** 10.1371/journal.pone.0068222

**Published:** 2013-07-09

**Authors:** Lei Chen, Yongchun Shen, Lian Liu, Xiaoou Li, Tao Wang, Fuqiang Wen

**Affiliations:** 1 Division of Pulmonary Diseases, State Key Laboratory of Biotherapy of China, West China Hospital, West China School of Medicine, Sichuan University, Chengdu, Sichuan, China; 2 Department of Respiratory Medicine, West China Hospital, West China School of Medicine, Sichuan University, Chengdu, Sichuan, China; National Jewish Health, United States of America

## Abstract

**Background:**

Interleukin (IL)-13, a T-helper type 2 cytokine, plays a critical role in the development of chronic obstructive pulmonary disease (COPD). This meta-analysis was performed to assess the association of IL-13 −1112 C/T promoter polymorphism with COPD susceptibility.

**Methods:**

Published case-control studies from Pubmed and China National Knowledge Infrastructure (CNKI) databases were retrieved. Data were extracted and pooled odds ratios (OR) with 95% confidence intervals (CI) were calculated.

**Results:**

Eight case-control studies in seven articles were included in this meta-analysis. Pooled effect size showed IL-13 −1112 C/T was associated with COPD susceptibility in a codominant genetic model (TT vs CT, OR: 1.82, 95% CI: 1.14–2.92 and TT vs CC, OR: 2.02, 95% CI: 1.10–3.72), indicating individuals with TT genotype had an increased risk for COPD compared with those with CT or CC genotype. According to ethnicity, results indicated IL-13 −1112 C/T was correlated with COPD susceptibility in Arabians (TT vs CT, OR: 2.94, 95% CI: 1.03–8.42 and TT vs CC, OR: 3.05, 95% CI: 1.08–8.59). Moreover, after excluding the study without Hardy-Weinberg equilibrium, the pooled results were robust and no publication bias was found in this study.

**Conclusions:**

This meta-analysis suggests IL-13 −1112 C/T promoter polymorphism is associated with the risk of COPD in Arabians.

## Introduction

Interleukin (IL)-13 is a T-helper type 2 (Th2) cytokine and its gene position is together with the IL-4 gene located within 15 kb on chromosome 5q31, a region associated with asthma, airway hyper-responsiveness and IgE levels [1]. Pulmonary expression of transgenic IL-13 in adult lungs resulted in a chronic obstructive pulmonary disease (COPD) phenotype with inflammation, mucus metaplasia and matrix metalloproteinase- and cathepsin-dependent emphysema [2]. Furthermore, increased IL-13 expression was detected in peripheral blood of COPD subjests and bronchial submucosa in smokers with chronic bronchitis [3], [4]. These data indicated a critical role of IL-13 in COPD.

Unfortunately, IL-13 gene was not selected as a candidate gene for COPD in recent genome-wide association studies (GWAS) [5]. The reasons could be i) most of the recent GWAS were performed in the Northern European populations [6]; ii) SNPs that possibly had significance but not to reach the genome-wide significant level might be covered in GWAS [7]. So, the lack of GWAS results do not preclude the involvement of polymorphisms in IL-13 gene with COPD. More importantly, van der Pouw Kraan et al firstly suggested an association of the changes from cytosine to thymine at −1112 C/T in a promoter region associated with altered regulation of IL-13 production in COPD [8]. Thereafter, a cluster of studies on the association of IL-13 −1112 C/T (also named −1055 C/T or −1111 C/T) [9] polymorphism with COPD susceptibility have been performed in the past decade [10]–[15]. Furthermore, recent studies have futher indicated subjects with extensive smoking exposure possessing the −1112 C/T allele developed worsening airflow obstruction [9], [16]. Therefore, IL-13 −1112 C/T promoter polymorphism may be a risk factor for COPD.

However, difference in ethnicity and sample size in individual studies may result in inconsistent results with lower statistical powers, and a meta-analysis could be a useful means to pool the independent statistical powers and thus achieve a quantitative understanding of the associations. Accordingly, in the present study, a meta-analysis was performed to determine IL-13 −1112 C/T promoter polymorphism and the risk of COPD.

## Methods

### Search Strategy

Literature search was conducted using Pubmed and China National Knowledge Infrastructure (CNKI) (http://www.cnki.net/) databases. The languages were limited to English and Chinese. The following search terms were utilized: interleukin 13 or IL 13, and gene polymorphism or polymorphism or variant, and chronic obstructive pulmonary disease or COPD. The PRISMA flow diagram (Figure S1) and checklist (Table S1) were available as supporting information.

### Data Extraction

Two independent reviewers collected the data according to an inclusion and exclusion critera. For inclusion in the meta-analysis, retrieved articles had to inform number of cases and controls, and number of individual genotype in cases and controls. Exlusion criteria in the meta-analysis were 1) not case-control genetic study, 2) duplicated report, 3) no useful data reported, 4) other IL-13 polymorphisms except −1112 C/T (also named −1055 C/T, or −1111 C/T). Unpublished data were not considered. Disagreement was resolved by discussion before reaching a consensus. If more than one article was published by the same group using the same cases, the study with higher sample size was selected.

### Statistical Analyses

Categorical variables were presented as odds ratio (OR) with 95% confidence interval (CI). CC, CT and TT are the genotypes of IL-13 −1112 C/T polymorphism. OR1, OR2 and OR3 were calculated as follows: 1) TT vs CC; 2) CT vs CC; 3) TT vs CT. These pairwise differences (OR1, OR2 and OR3) were used to indicate the most appropriate genetic model as follows: if OR1 = OR3≠1 and OR2 = 1, then a recessive model was suggested; if OR1 = OR2≠1 and OR3 = 1, then a dominant model was suggested; if OR2 = 1/OR3≠1 and OR1 = 1, then a complete overdominant model was suggested; if OR1>OR2>1 and OR1>OR3>1 (or OR1<OR2<1 and OR1<OR3<1), then a codominant model was suggested. Once the best genetic model was identified, this model was used to collapse the three genotypes into two groups (except a codominant model) and to pool the results again [17]. Pooled ORs with 95% CI were calculated and p<0.05 was accepted with statistical significance. Heterogeneity was checked by the Q test. Meta-analysis was done with the fixed-effects model when there was no heterogeneity (p≥0.1). Otherwise, the random-effects model was used. Subgroup analysis was performed by enthinity to assess the effect of possible clinical heterogeneity on the summary ORs. Pearson’s *χ* ^2^ test was used to determine whether the observed frequencies of genotypes in controls conformed to the Hardy–Weinberg equilibrium (HWE). Studies with controls that depart from HWE (*p*<0.05) were subjected to a sensitivity analysis in order to check the consistency of the overall effect size. Funnel plots, as well as the Begg’s rank correlation test and Egger’s linear regression test, was used to inspect the potential publication bias, and p<0.05 was considered significant publication bias. All analyses were conducted using Revman 5.0 (Oxford, UK, The Cochrane Collaboration) and Stata 11.0 (StataCorp LP, College Station, TX, USA).

## Results

### Studies Included in the Meta-analysis

**Figure 1:**
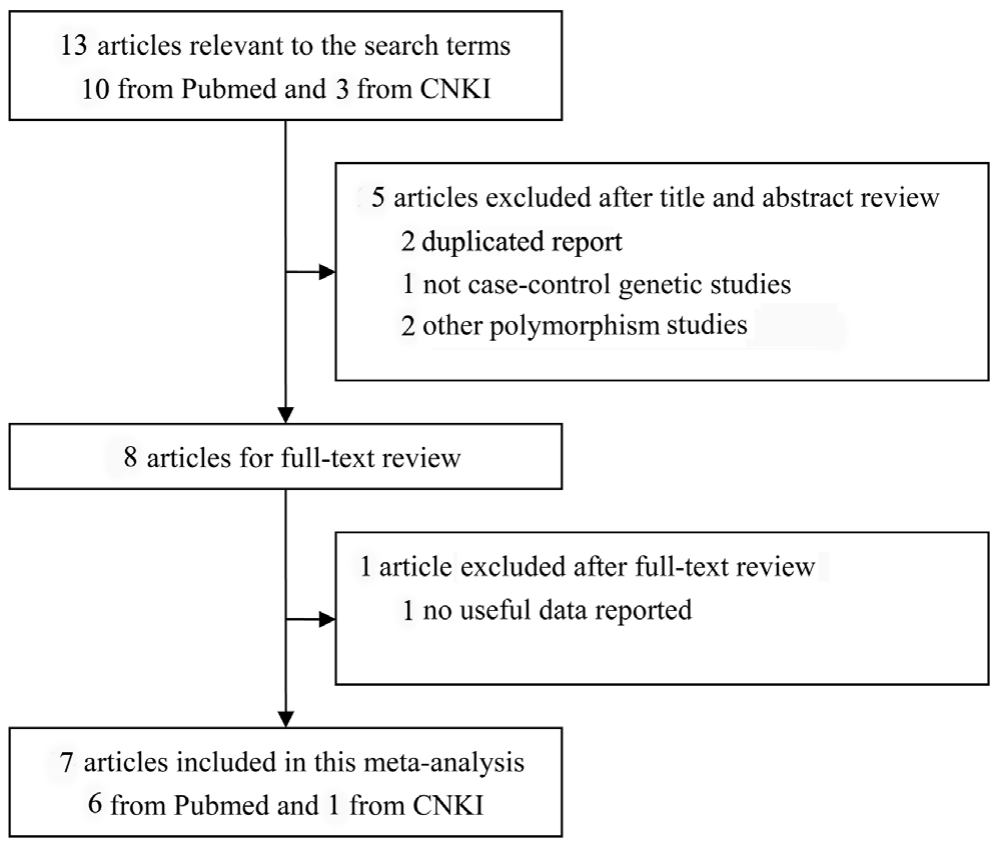
Flow diagram of search process.

Thirteen studies were relevant to the search terms. After reviewing the titles, abstracts and articles, six studies were excluded and thus seven studies with 1319 cases and 831 controls matched the inclusion criteria (**Figure 1**). Of the seven included studies, six were published in English, and one was published in Chinese. These studies were carried out in China, Taiwan, Japan, Egypt, United Kingdom and Netherland. Noticeablely, the study by Hegab et al [10] was performed in two ethnic populations (Japan and Egypt). The main features of the studies included in this meta-analysis were presented in **Table 1**.

**Table 1:** Main characteristics of included studies.

Ref	Country	Race	Genotyping	Source of control	Case (N)				Control (N)				
					Total	TT	CT	CC	Total	TT	CT	CC	HWE (*p*)
[8]	Netherland	Caucasian	PCR+RFLP	Healthy population	151	9	62	80	177	5	44	128	0.877
[10]	Japan	Asian	PCR+RFLP	Healthy smoker	88	1	36	51	61	1	21	39	0.620
[10]	Egypt	Arabian	PCR+RFLP	Healthy smoker	105	8	35	62	71	2	28	41	0.551
[11]	China	Asian	PCR+RFLP	Healthy population	111	13	6	92	97	13	14	70	0.000
[12]	Taiwan	Asian	PCR+RFLP	Healthy smoker	85	9	14	62	72	0	2	70	0.993
[13]	UK	Caucasian	KASPar assay	Healthy smoker	564	22	147	395	173	6	46	121	0.825
[14]	China	Uygur	ABI sequencer	Healthy population	140	15	47	78	140	5	35	100	0.687
[15]	Egypt	Arabian	ABI sequencer	Healthy population	75	15	29	31	40	3	16	21	0.999

HWE: Hardy-Weinberg equilibrium; N: number; PCR: polymerase chain reaction; Ref: reference; RFLP: restriction fragment length polymorphism; UK: United Kingdom.

### Quantitative Synthesis

**Figure 2:**
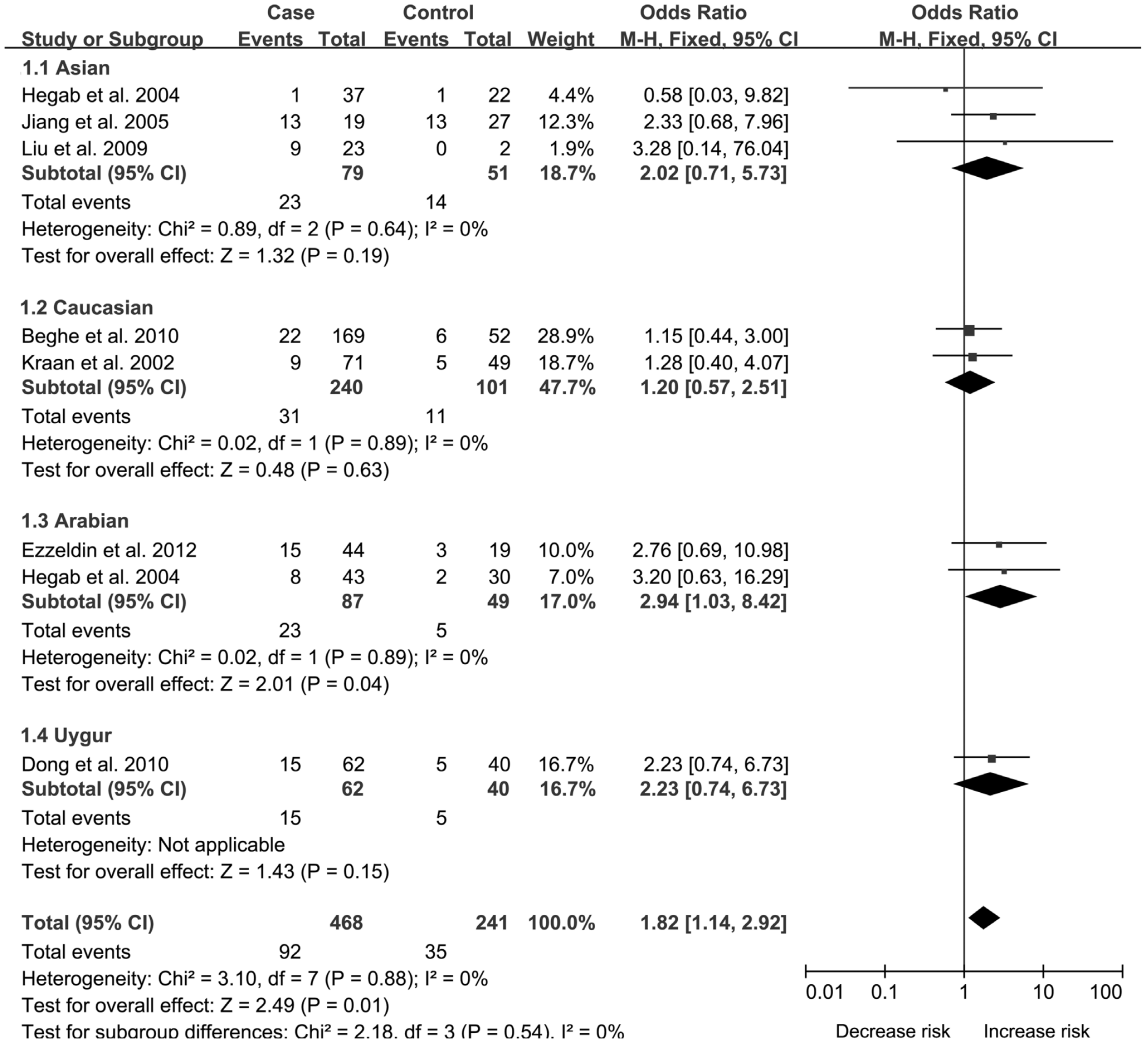
Forest plots of OR with 95% CI for the association of IL-13−1112 C/T and COPD risk subanalyzed by enthnicity in TT vs CT.

**Figure 3:**
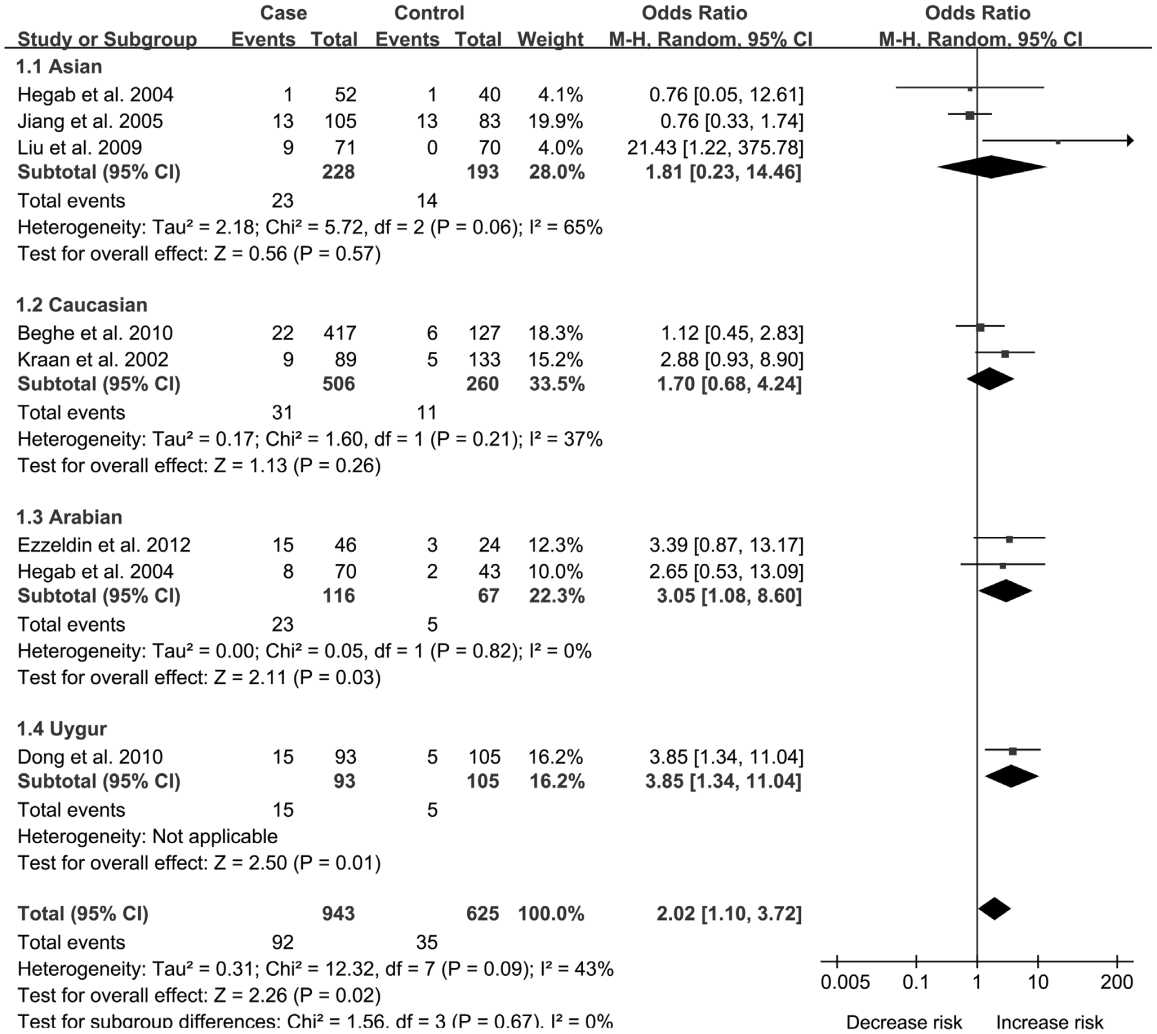
Forest plots of OR with 95% CI for the association of IL-13−1112 C/T and COPD risk subanalyzed by enthnicity in TT vs CC.

In order to indicate the most appropriate genetic model, OR1 (TT vs CC), OR2 (CT vs CC) and OR3 (TT vs CT) were calculated. Results showed OR1 = 2.02, OR2 = 1.28 and OR3 = 1.82 respectively, suggesting a codominant genetic model (TT vs CT and TT vs CC). Using a codominant genetic model, pooled effect size showed an association of IL-13 −1112 C/T with the risk of COPD (TT vs CT, OR: 1.82, 95% CI: 1.14–2.92, **Figure 2** and TT vs CC, OR: 2.02, 95% CI: 1.10–3.72, **Figure 3**), which indicated individuals with TT genotype had a higher risk for COPD than those with CT or CC genotypes. In the subgroup analysis by ethnicity, results indicated IL-13 −1112 C/T was correlated with COPD susceptibility in Arabians (TT vs CT, OR: 2.94, 95% CI: 1.03–8.42 and TT vs CC, OR: 3.05, 95% CI: 1.08–8.59).

### Test of Heterogeneity

Significant heterogeneity was revealed between all studies in TT vs CC comparison (I^2^ = 43%, p = 0.09), but not TT vs CT comparison (I^2^ = 0%, p = 0.88), and the source of heterogeneity for TT vs CC comparison was detected by ethnicity. When stratified by ethnicity, no heterogeneity was observed in the studies on TT vs CC comparison in Caucasians and Arabians, suggesting heterogeneity between the studies in Asians and Uygur contributed significantly to the overall heterogeneity in TT vs CC comparison.

### Sensitivity Analyses

In the present meta-analysis, only one study [11] was lack of HWE, which had a potential to influence the robustness of the present meta-analysis. After exclusion of this study, the pattern of the pooled effect size persisted significance in both TT vs CC and TT vs CT comparisons (data not shown), while the overall heterogeneity in TT vs CC comparison was altered significantly (I^2^ = 11%, p = 0.34), indicating this study contributed more to the overall heterogeneity in TT vs CC comparison.

### Publication Bias

**Figure 4:**
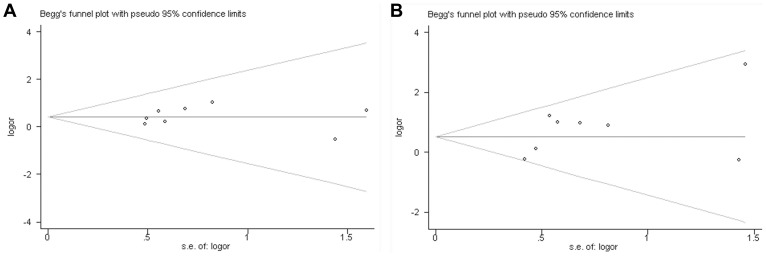
Begg’s funnel plot for evaluation of publication bias in the included studies on the association of IL-13−1112 C/T and COPD risk (Panel A: TT vs CT and Panel B: TT vs CC).

The funnel plots showed no sigificant asymmetry in this meta-analysis (**Figure 4**). Moreover, publication bias was not suggested by Begg’s rank correlation test (TT vs CT: p = 0.386, TT vs CC: p = 0.386) and Egger’s linear regression test (TT vs CT: p = 0.804; TT vs CC: p = 0.185).

## Discussion

IL-13, a Th2 cytokine, contributes to the development of COPD. Recently, association of IL-13 −1112 C/T promoter polymorphism with COPD risk caught more attention, owing to its potential in regulation of IL-13 production and correlation with cigarette smoke-induced airflow limitation [8], [9], [16].

In this meta-analysis, IL-13 −1112 C/T was found to be correlated with COPD in a codominant model (the most appropriate genetic model), indicating individuals with TT genotype had a higher risk for COPD than those with CT or CC genotype, which is consistent with the reports by Sadeghnejad et al. Interestingly, no heterogeneity was revealed between the studies in TT vs CT comparison, however, there was significant heterogeneity between the studies in TT vs CC comparison. To identify the source of heterogeneity in TT vs CC comparison, subgroup analysis was performed according to ethnicity. No heterogeneity was observed in the studies for TT vs CC comparison in Caucasians and Arabians, suggesting heterogeneity between the studies in Asians and Uygur contributed significantly to the overall heterogeneity. Futher results indicated IL-13 −1112 C/T was correlated with COPD susceptibility in Arabians but not Caucasians with homogeneity in both TT vs CT and TT vs CC comparisons. After exclusion of the study without HWE by Jiang et al [11], the pattern of the pooled effect size persisted significance in both TT vs CT and TT vs CC comparisons, while the overall heterogeneity in TT vs CC comparison was altered significantly, indicative of this study might contribute more to the overall heterogeneity than other studies in Asians and Uygur in TT vs CC comparison. Publication bias was not suggested in the present study, possibly owing to the deliberate search strategy and data extraction.

However, some limitations should be considered in this meta-analysis. First, large sample size studies were lacked and the pooled statistical powers could not be very enough. Second, the pooled estimates were not based on adjustment by confused factors, such as sex, age, smoking history. Third, lack of the original data in the studies limited our further analysis of the potential interactions between gene and gene, or gene and environment, which might modulate COPD risk.

In conclusion, although the pooled estimates should be interpreted with caution, our meta-analysis suggests that IL-13 −1112 C/T promoter polymorphism is associated with the risk of COPD in Arabians. However, large sample size studies with unbiased genotyping methods, standardized defined COPD cases and matched controls in different populations, and more detailed data about individual and environment are warranted.

## Supporting Information

Figure S1 **PRISMA flow diagram.**
(DOC)

Table S1 **PRISMA checklist.**
(DOC)
