# Supplementary material for: Interleukin-13 −1112 C/T Promoter Polymorphism Confers Risk for COPD: A Meta-Analysis
Source: PLoS One. 2013 Jul 9;8(7):e68222. doi: 10.1371/journal.pone.0068222 (PMC3706578; doi:10.1371/journal.pone.0068222)
Supplement: Figure S1 — PRISMA flow diagram. (DOC) [file pone.0068222.s001.doc]

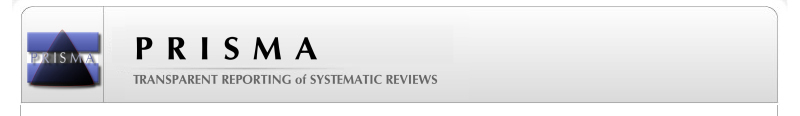
**PRISMA Flow Diagram**

**Screening**

**Included**

**Eligibility**

**Identification**

Records identified through database searching
(n =13)

Additional records identified through other sources
(n =0)

Records after duplicates removed
(n =11)

Records screened
(n = 11 )

Records excluded
(n = 3)

Full-text articles assessed for eligibility
(n = 8)

Full-text articles excluded, with reasons
(n =1)

Studies included in qualitative synthesis
(n =7)

Studies included in quantitative synthesis (meta-analysis)
(n = 7 )
